# ALK inhibitors in cancer: mechanisms of resistance and therapeutic management strategies

**DOI:** 10.20517/cdr.2024.25

**Published:** 2024-05-23

**Authors:** Darin Poei, Sana Ali, Shirley Ye, Robert Hsu

**Affiliations:** ^1^Department of Internal Medicine, University of Southern California, Los Angeles, CA 90033, USA.; ^2^Division of Medical Oncology, University of Southern California Norris Comprehensive Cancer Center, Los Angeles, CA 90033, USA.

**Keywords:** NSCLC, ALK TKI, acquired resistance, alectinib, crizotinib, lorlatinib, ceritinib, brigatinib

## Abstract

Anaplastic lymphoma kinase (*ALK*) gene rearrangements have been identified as potent oncogenic drivers in several malignancies, including non-small cell lung cancer (NSCLC). The discovery of ALK inhibition using a tyrosine kinase inhibitor (TKI) has dramatically improved the outcomes of patients with ALK-mutated NSCLC. However, the emergence of intrinsic and acquired resistance inevitably occurs with ALK TKI use. This review describes the molecular mechanisms of ALK TKI resistance and discusses management strategies to overcome therapeutic resistance.

## INTRODUCTION

The evaluation and management of non-small cell lung cancer (NSCLC) have dramatically evolved since the discovery of oncogenic driver mutations, such as anaplastic lymphoma kinase (ALK)^[1,2]^. First described in anaplastic large-cell lymphoma in 2004, the *ALK* gene encodes a highly conserved receptor tyrosine kinase (RTK) in the insulin receptor superfamily^[3,4]^. Structurally, ALK is made up of an extracellular domain, a single-pass transmembrane helix, and an intracellular tyrosine kinase domain. Upon binding of endogenous ALKAL1 and ALKAL2 ligands to the extracellular domains, dimerization and autophosphorylation of the intracellular kinase domains lead to the activation of downstream signaling pathways critical for cellular proliferation, survival, and differentiation^[5,6]^. In several cancers, structural ALK rearrangements whereby the kinase domain-encoding region at the 3’end is fused to various partner genes located at the 5’end have been identified as key oncogenic drivers^[5]^. Perhaps most commonly recognized is the EML4-ALK fusion, which was first discovered by Soda *et al*. in a small cohort of Japanese patients with NSCLC. Within EML4-ALK, there are at least 12 distinct variants, with the two most common being variant 1 (E13;A20) and variant 3 (E6/b;A20)^[7,8]^. To date, more than 90 ALK rearrangements have been identified across 3-7% of all patients diagnosed with advanced NSCLC^[9]^. ALK rearrangements in NSCLC have been most commonly identified in adenocarcinoma, but have also been seen in squamous cell carcinoma and lymphoepithelioma-like carcinoma^[10-12]^.

**Figure 1:**
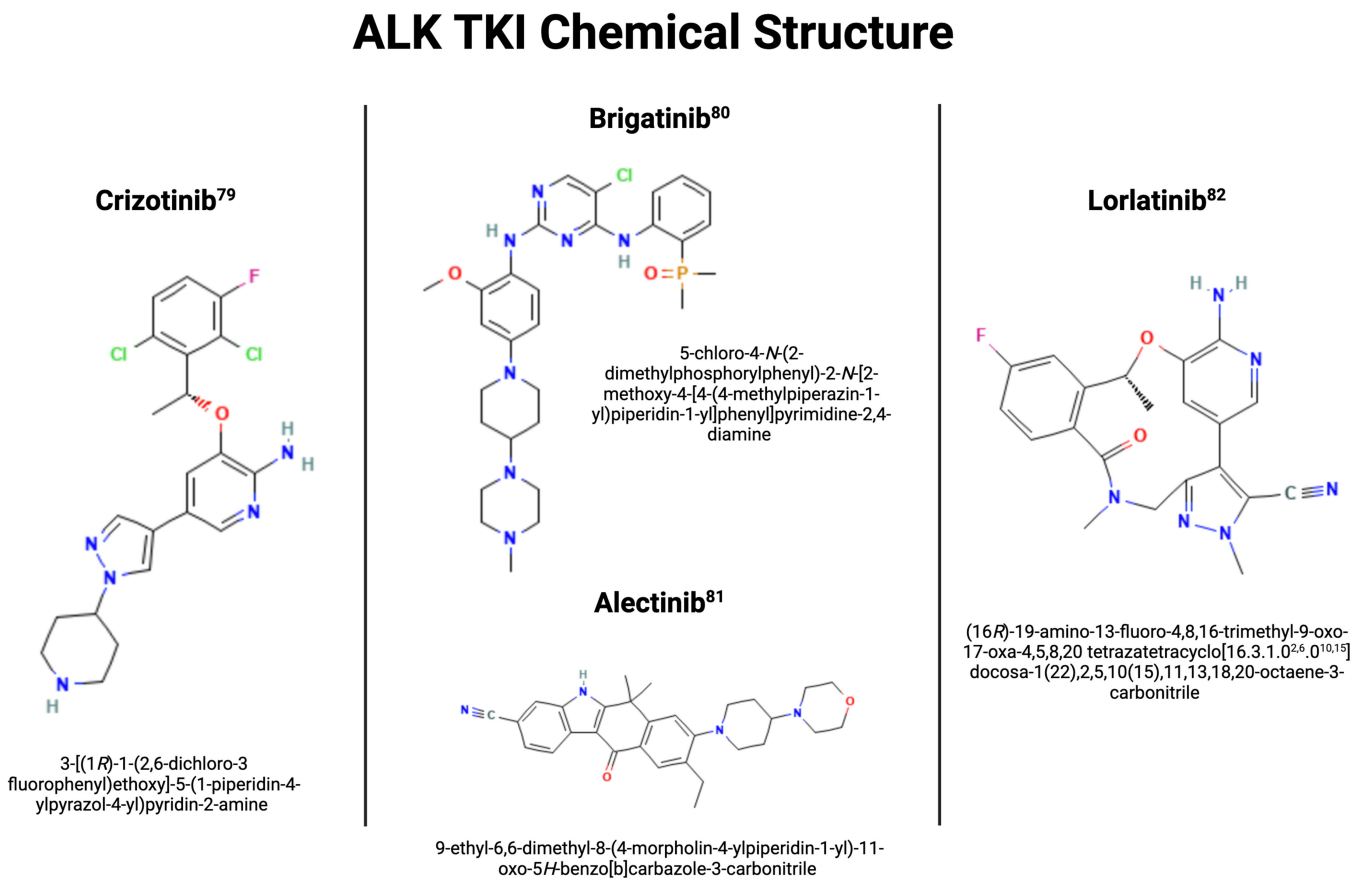
ALK TKI Chemical Structure^[37]^. Created by BioRender.com. Authors were granted a license to use BioRender content with copyright permission. ALK: Anaplastic lymphoma kinase; TKI: tyrosine kinase inhibitor.

Shortly after the discovery of ALK rearrangements, it was determined that ALK rearrangements confer significant sensitivity to ALK inhibition using a tyrosine kinase inhibitor (TKI). Crizotinib, a first-in-class ALK/ROS1/MET inhibitor, was approved by the US Food and Drug Administration (FDA) for advanced, ALK-altered NSCLC in 2011. In two randomized phase III trials, crizotinib was associated with significantly higher PFS (10.9 months *vs*. 7.0 months in the first-line PROFILE 1014 trial and 7.7 *vs*. 3.0 months in the PROFILE 1007 trial involving pre-treated patients) and objective response rate (ORR) compared to first- and second-line cytotoxic chemotherapy, resulting in its approval as frontline therapy in patients with advanced, ALK-positive NSCLC^[9,13]^. Since then, several next-generation ALK TKI have been developed with increasing on-target potency and central nervous system (CNS) penetration. There are currently five ALK TKI approved by the US FDA for the treatment of advanced, ALK-altered NSCLC [Table 1]: crizotinib (first-generation), ceritinib, alectinib, brigatinib (second-generation), and lorlatinib (third-generation) [Figure 1]^[37-41]^. Despite these advances, management remains challenging as all patients with advanced, ALK-positive NSCLC treated with ALK TKI therapy eventually develop disease progression due to various mechanisms of drug resistance^[42]^. In this review, we provide an overview of molecular mechanisms of ALK TKI resistance and therapeutic strategies to overcome resistance.

**Table 1:** Summary of FDA-approved ALK inhibitors

**ALK TKI**	**Phase**	**Trial**	**Treatment**	**TKI dose**	**PFS (months) (95% CI)**	**ORR% (95% CI)**	**Ref.**
**1st generation**							
Crizotinib	I	PROFILE 1001	Single arm; crizotinib naive	Crizotinib 250 mg BID	9.7 (7.7-12.8)	60.8 (52.3-68.9)	Camidge *et al*., 2012^[14]^
	II	PROFILE 1005	Single arm; recurrent disease after ≥ 1 chemotherapy	Crizotinib 250 mg BID	8.1 (6.8-9.7)	60.0 (53.6-65.9)	Kim *et al*., 2012^[15]^
	III	PROFILE 1007	*Vs*. chemotherapy (pemetrexed or docetaxel); second line after one prior platinum-based regimen	Crizotinib 250 mg BID	7.7 (6.0-8.8) *vs*. 3.0 (2.6-4.3)	65 (58-72) *vs*. 19.5 (14-26)	Shaw *et al*., 2013^[9]^
	III	PROFILE 1014	*Vs.* chemotherapy (pemetrexed and cisplatin or pemetrexed and carboplatin); first line	Crizotinib 250 mg BID	10.9 (8.3-13.9) *vs*. 7.0 (6.8-8.2)	74 (67-81) *vs*. 45 (37-53)	Solomon *et al*., 2014^[13]^
	III	PROFILE 1029	*Vs.* chemotherapy (pemetrexed and cisplatin or pemetrexed and carboplatin); first line	Crizotinib 250 mg BID	11.1 (8.3-12.6) *vs*. 6.8 (5.7-7.0)	87.5 (79.6-93.2) *vs*. 45.6 (35.8-55.7)	Wu *et al*., 2018^[16]^
**2nd generation**							
Ceretinib	I	ASCEND-1	Single arm; refractory disease despite standard therapy	Ceretinib 750 mg daily	18.4 (11.1 - NE) in ALKi naive *vs*. 6.9 (5.6-8.7) in ALKi pretreated	72 (61-82) in ALKi naive *vs*. 56 (49-64) in ALKi pretreated	Kim *et al*., 2016^[17]^
	II	ASCEND-2	Single arm; recurrent disease after ≥ 1 chemotherapy who progressed ≤ 30 days from last treatment with crizotinib	Ceretinib 750 mg daily	5.7 (5.4-7.6)	38.6 (30.5-47.2)	Crinò *et al*., 2016^[18]^
	II	ASCEND-3	Single arm; ALK inhibitor naive	Ceretinib 750 mg daily	11.1 (9.3 - NE)	63.7 (54.6-72.2)	Felip *et al*., 2015^[19]^
	III	ASCEND-4	*Vs.* chemotherapy (pemetrexed and cisplatin or pemetrexed and carboplatin); first line	Ceretinib 750 mg daily	16.6 (12.6-27.2) *vs*. 8.1 (5.8-11.1)		Soria *et al*., 2017^[20]^
	III	ASCEND-5	*Vs*. chemotherapy (pemetrexed or docetaxel); progression after chemotherapy and crizotinib	Ceretinib 750 mg daily	5.4 (4.1-6.9) *vs*. 1.6 (1.4-2.8)	39.1 (30.2-48.7) *vs*. 6.9 (3.0-13.1)	Shaw *et al*., 2017^[21]^
	I/II	ASCEND-6	Single arm; progression on crizotinib	Ceretinib 750 mg daily	5.7 (5.4-7.5)	40.8 (31.2-50.9)	Zhang *et al*., 2016^[22]^
	I	ASCEND-8	*Vs*. ceretinib 450 mg daily and ceretinib 600 mg daily	Ceretinib 750 mg daily	450 mg: NE (11.2 - NE) *vs*. 600 mg: 20.7 (15.8 - NE) *vs*. 750 mg: 15.4 (8.3 - NE)	450 mg: 78.1 (66.9-86.9) *vs*. 600 mg: 72.5 (58.3-84.1) *vs*. 750 mg: 75.7 (64.3-84.9)	Cho *et al*., 2017^[23]^
Alectinib	I/II	AF-001JP	Single arm; ALK inhibitor naive	Alectinib 300 mg BID		93.5 (82.1-98.6)	Seto *et al*., 2013^[24]^
	II	NP28761	Single arm; progression on crizotinib	Alectinib 600 mg BID		47.8 (35.6-60.2)	Gandhi *et al*., 2015^[25]^
	II	NP28673	Single arm; progression on crizotinib	Alectinib 600 mg BID		49.2 (40.0-58.4)	Ou *et al*., 2015^[26]^
	III	ALEX	*Vs*. crizotinib; first line	Alectinib 600 mg BID		82.9 (76.0-88.5) *vs*. 75.5 (67.8-82.1)	Peters *et al*., 2017^[27]^
	III	ALUR	*Vs*. chemotherapy (pemetrexed or docetaxel); progression after chemotherapy and crizotinib	Alectinib 600 mg BID	7.1 (6.3-10.8) *vs*. 1.6 (1.3-4.1)		Novello *et al*., 2018^[28]^
	III	J-ALEX	*Vs*. crizotinib; first line	Alectinib 300 mg BID	NYR (20.3 - NE) *vs*. 10.2 (8.2-12.0)		Hida *et al*., 2017^[29]^
	III	ALESIA	*Vs*. crizotinib; first line	Alectinib 600 mg BID	NE *vs*. 11.1		Zhou *et al*., 2019^[30]^
Brigatinib	I/II	ALTA	*Vs*. brigatinib 90 mg daily; progression after crizotinib	Brigatinib 90 mg daily for 7 days, then 180 mg daily	9.2 *vs*. 15.6	48 *vs*. 53	Hochmair *et al*., 2017^[31]^
	III	ALTA-1L	*Vs*. crizotinib; first line	Brigatinib 90 mg daily for 7 days, then 180 mg daily		71 (62-78) *vs*. 60 (51-68)	Camidge *et al*., 2018^[32]^
	II	J-ALTA	Single arm; progression on alectinib and/or crizotinib	Brigatinib 90 mg daily for 7 days, then 180 mg daily	7.3 (3.7-12.9)	34 (21-49)	Yoshida *et al*., 2023^[33]^
	II	ALTA-2	Single arm; progression on alectinib or ceretinib	Brigatinib 90 mg daily for 7 days, then 180 mg daily	3.8 (3.5-5.8)	26.2 (18.0-35.8)	Kim *et al*., 2021^[34]^
	III	ALTA-3	*Vs*. alectinib; progression on crizotinib	Brigatinib 90 mg daily for 7 days, then 180 mg daily	19.3 (15.7 - not reached) *vs*. 19.2 (12.9 - not reached)	52 (43-61) *vs*. 61 (52-70)	Yang *et al*., 2023^[35]^
**3rd generation**							
Lorlatinib	III	CROWN	*Vs*. crizotinib; first line	Lorlatinib 100 mg daily	78 (70-84) *vs*. 39% (30-48)	76 (68-83) *vs*. 58% (49-66)	Shaw *et al*., 2020^[36]^

FDA: Food and Drug Administration; ALK: anaplastic lymphoma kinase; TKI: tyrosine kinase inhibitor; BID: twice a day; PFS: progression-free survival; 95% CI: 95% confidence interval; ORR: objective response rate; NE: not estimated; ALKi: ALK inhibitor; NYR: not yet reached at the time of data analysis.

## MOLECULAR MECHANISMS OF ALK INHIBITOR RESISTANCE

Molecular mechanisms of resistance to ALK TKI therapy can be broadly classified as intrinsic (i.e., primary) or acquired (i.e., secondary). Intrinsic resistance is defined as a de novo lack of response to treatment and is considered when progression is noted within 3 months of ALK TKI initiation^[42]^. Intrinsic resistance to ALK TKI is uncommon and has been observed in 4%-10% of patients with ALK-positive NSCLC^[36,43]^. Acquired resistance refers to disease progression after a period of initial clinical benefit. Molecular mechanisms of acquired resistance can be further subdivided into ALK-dependent (“on-target”) or ALK-independent (“off-target”). Tumor cells with on-target resistance retain their dependence on ALK, while those with off-target resistance utilize alternative ALK-independent pathways to support proliferation^[8]^.

### Mechanisms of acquired ALK-dependent or “on-target” resistance

ALK-dependent or “on-target” resistance refers to single or compound mutations within the ALK tyrosine kinase domain, which induce resistance by way of direct steric hindrance of TKI binding, alteration in protein kinase conformation, and/or changes in ATP binding^[44-46]^. On-target resistance accounts for 30%-40% of all known resistance mechanisms^[47]^.

The most commonly acquired resistance mechanism to ALK TKI is G1202R, which is seen in nearly half of patients receiving brigatinib, over 20% of the patients receiving ceritinib and alectinib, and 10% of patients receiving crizotinib^[44,48]^. Other commonly acquired resistance mutations in ALK TKI include L1196M gatekeeper mutations. Initially identified in a cell line model with crizotinib resistance, these mutations have since been implicated in patients receiving both ceritinib and brigatinib^[49]^. Non-G1202R acquired mutations comprise 40%-65% of acquired resistance mechanisms in second-generation ALK TKI. Additional resistance mechanisms implicated in alectinib resistance include I1171T and V1180L, while other brigatinib resistance mechanisms include D1203N, S1206Y/C, and E1210K. Resistance mechanisms implicated in patients taking ceritinib include C1156Y, I1171T, V1180L, L1196M, and D1203N^[44,47,48]^. In crizotinib, the most common patterns of resistance are L1196M, G1269A, and G1202R mutations, which comprise 20-30% of all crizotinib-resistant cases [Table 2]^[42]^. Specific molecular mechanisms of resistance include alteration of the ATP-binding pocket of ALK, affecting the residue of the N-terminus, and solvent front mutations^[54-56]^.

**Table 2:** ALK inhibitors and associated mutations

**ALK TKI**	**Mutations associated with resistance**	**Mutations associated with susceptibility**	**Potential methods to overcome resistance**
**1st generation**			
Crizotinib	On-target mutations: G2032R, I1151Tins, L1152P/R, C1156T/Y, I1171N/S, F1174C/L/V, V1180L, L1196M, L1198P, D1203N, S1206C/Y, E1210K, F1245C/V, G1269A, G1269S, G1202R Off-target mutations: EGFR tyrosine phosphorylation ALK amplification EMT Myc amplification Overexpression of P-glycoprotein HER2/3 receptors	On-target mutations: L1198F, G1123S, C1156Y + L1198F	Next-generation ALK TKI Combination with HSP90 inhibitor, induces loss of EML4-ALK expression^[49]^ Combination with Silibin, a STAT3 inhibitor^[50]^
**2nd generation**			
Ceritinib	On-target mutations: G1202R, G1128A, G1269A, I117IT + E1210K, L1152P/R, C1156T/Y, L1198F, D1203N, E1210K, V1180L, L1196M, D1203N, G1202R + G1269A, G1202R + L1196M, F1176 C/L, G1202R + F1174V, I1171N + F1174L, I1171N + L1198H Off-target mutations: EMT Overexpression of P-glycoprotein *c-MET* gene amplification	On-target mutations: I1171N/T, G1269A/S, V1180L, S1206C/Y, I1171N + G1269A, I1171N + L1196M	Alternate/Next-generation ALK TKI Combination with Afatinib, an EGFR TKI^[51]^
Alectinib	On-target mutations: G1202R, I1171T/S/N, V1180L, W1295C, L1196M, G1202L, L1198F, D1203N, E1210K, F1245C Off-target mutations: EGFR pathway activation GAB1 ABCC11	On-target mutations: C1156Y/T, G1269A I1151Tins, L1152P, L1152R, F1174C/L/V, S1206C/Y, G1269A/S	Alternate/Next-generation ALK TKI Combination with metformin Combination with Afatinib^[51]^
Brigatinib	On-target mutations: G1202R, E1210K, S1206Y/C, L1198F, D1203N, E1407K, F1245C	On-target mutations: C1156Y/T, G1123S, I1151Tins, L1152P/R, I1171N/T/S, F1174C/L/V, V1180L, L1196M, S1206Y, G1269A/S	Alternate/Next-generation ALK TKI
**3rd generation**			
Lorlatinib	On-target mutations: L1198F, G2032R, D1203N, G1123D, C1156Y + L1198F, G1202R + L1196M, I1171N + D1203N Off-target mutations: MET amplification	On-target mutations: L1196M, G1202R, G1269A, G1123S, I1151Tins, L1152P/R, C1156T/Y I1171T, F1174C/L/V, V1180L, S1206C/Y, E1210K, F1245C, G1269A/S	Potential 4th generation agents (e.g., Zotizalkib)^[52]^ Repotrectinib, a ROS1 TKI^[52]^ Gilteritinib, a FLT3 inhibitor^[53]^

ALK: Anaplastic lymphoma kinase; TKI: tyrosine kinase inhibitor; EGFR: epidermal growth factor receptor; EMT: epithelial-to-mesenchymal transition; ABCC11: ATP- binding domain C-member 11.

ALK amplification has also been implicated in the development of ALK-dependent resistance, notably in acquired resistance to crizotinib, although at a much lower frequency. In a series of 18 patients with acquired resistance to crizotinib, only one patient demonstrated high-level ALK amplification without accompanying ALK mutation^[47]^. In another series of 11 patients with acquired resistance to crizotinib, two patients demonstrated ALK copy number gain, although one had concurrent ALK resistance mutation^[57]^. ALK amplification has not yet been identified as a mechanism of acquired resistance in second- or third-generation ALK TKI, suggesting that it may not be a clinically relevant mechanism for more potent ALK inhibitors^[44]^.

Another area of consideration is the acquisition of compound or double mutations, particularly with the sequential use of different ALK inhibitors during treatment. ALK C1156Y + L1198F double mutants have been shown to confer resistance to lorlatinib, but sensitivity to crizotinib^[58]^. In a preclinical study, mice that were previously treated with alectinib and lorlatinib developed I1171N + L1256F mutations but demonstrated anti-tumor efficacy when retreated with alectinib. Ceritinib was found to be effective in I1171N + G1269A and I1171N + L1196M mutations but resistant to G1202R + G1269A or G1202R + L1196M mutations [Table 2]^[58]^. These combination mutations highlight the need to continue monitoring for acquired mutations upon disease progression with ALK TKI and to consider rechallenge of ALK TKI based upon the acquired compound mutations.

### Mechanisms of acquired ALK-independent or “off-target” resistance

When patients who develop disease progression following the use of ALK TKI are not found to harbor ALK mutations or amplifications, they are classified as having developed ALK-independent resistance. ALK-independent resistance accounts for 50% of patients with disease progression on second-generation ALK TKI^[59]^. Several off-target mechanisms have also been identified, including bypass signaling pathways, histologic transformation, and drug efflux pumps.

#### Bypass signaling pathways

ALK-independent resistance can develop via bypass signaling pathways, which are activated by changes in protein expression, genetic composition, and/or feedback signaling. The first bypass signaling pathway was identified in the setting of crizotinib resistance^[55]^. When compared to crizotinib-sensitive cells, crizotinib-resistant cells were associated with higher levels of epidermal growth factor receptor (EGFR) tyrosine phosphorylation, which led to the activation of downstream signal pathways despite the absence of an EGFR mutation^[47]^. There are other receptor tyrosine kinases including HER2/3, which have also been identified as validated bypass signaling pathways in ALK-independent resistance [Table 2]^[60]^.

In addition, the use of intracellular signaling via the MAPK pathway has been heavily implicated in ALK-independent resistance. Mutations of various members of this pathway, including KRAS, MEK, and MAP2K1, have been shown to increase resistance against ALK TKI^[61]^. This mechanism has led researchers to evaluate the use of ALK and MEK blockade to combat this form of resistance^[61]^. It was observed that inhibition of MEK increased the efficacy of ALK inhibition by decreasing MAPK signaling.

Another mechanism of bypass signaling is elevated hepatocyte growth factor expression and MET amplification, which has been identified in EGFR-mutated patients^[62,63]^. The role of MET amplification in off-target resistance was not identified until later because crizotinib confers dual MET and ALK inhibition. Later generations of ALK TKI do not exhibit MET inhibition, supporting the use of drug combinations targeting ALK and MET^[64]^. This has culminated in the design of a new clinical trial (NCT04292119), which is currently evaluating the role of various combination therapies, including ALK, MET, and MEK inhibitors. Another target is SH2 containing protein tyrosine phosphatase 2 (SHP2), which activates downstream tyrosine kinases by ALK bypass signaling^[65]^. It has been shown that inhibition of SHP2 with SHP099 and ceritinib halted the proliferation of resistant cell lines^[65]^. Another notable target in the HGF/MET signaling pathway is GAB1, which has been shown to lead to alectinib resistance. A study showed that metformin in combination with alectinib may help overcome alectinib resistance [Table 2]^[66]^.

Other targets for bypass signaling pathways include mast/stem cell growth factor receptor (KIT) and platelet-derived growth factor receptor alpha (PDGFRA) amplification. PDGFRA amplification has been associated with worsened PFS with crizotinib in patients with high c-KIT^[47,67]^, IGF-1R-IRS-1 pathway^[68,69]^, BRAF V600E mutation^[70]^, and Yes-associated protein 1 (YAP1). YAP1 has been shown to mediate survival in cancer cells that harbor the ALK rearrangement when they are treated with alectinib^[71]^. There are many targets involved in alternative bypass signaling pathways, which need to be further explored to provide a better understanding of this mechanism.

Despite the implication of bypass signaling pathways in off-target resistance, TKI-resistant cells may still rely on the *ALK* gene for cell signaling. This has been demonstrated in the clinical setting when patients develop disease flares following discontinuation of ALK TKI. Given the ongoing discovery of new bypass targets in patients with ALK TKI resistance, it will be important to continue expanding our understanding of this mechanism moving forward^[72]^.

#### Histologic transformation

The histologic transformation of cancer cells is another mechanism of ALK-independent resistance. Upon transformation of cancer cells into different histologic subtypes, the loss of driver mutations leading to oncogenesis results in ALK-independent resistance. In epithelial-to-mesenchymal transition (EMT), cancer cells transform from epithelial expression to mesenchymal expression. EMT is highly dynamic in cancer cells and frequently occurs as an incomplete process, and mesenchymal cells can revert to the epithelial state through mesenchymal-to-epithelial transition. Transcriptional factors with SNAI1 and SNAI2, ZEB1 and ZEB2, and TWIST, along with non-coding RNAs and epigenetic process, are drivers of this process^[73]^. The exact mechanism by which this confers resistance to ALK TKI remains unclear^[74]^. In one series of 12 patients with ceritinib resistance, EMT was identified in five cases, although three had concurrent ALK-dependent mutations^[44]^. It is thought that in these rare cases of transformation, there could be a co-existence of an ALK mutation and the presence of EMT mechanism. For example, a patient with crizotinib resistance was found to have an ALKL1196M resistance mutation and the EMT mechanism. It was later observed that the ALK G1202 mutation led to ceritinib resistance due to the induction of EMT mechanism via the STAT3/Slug pathway^[75]^. This finding may help researchers uncover the role of EMT in creating ALK TKI resistance. Another form of histologic transformation is the conversion of adenocarcinoma to small-cell lung cancer following the initiation of ALK TKI. This is a rare phenomenon that has been observed in case reports^[76,77]^. During the transformation to small cell lung cancer, it is thought that the acquisition of RB loss and TP53 deletion and other genetic alterations leads to resistance against ALK-targeted therapy^[78]^. Other notable markers seen on histological transformation include CD133, Bcl-2, and SOX2, which demonstrate that tumor stem cells may play a role in histologic transformation^[79]^.

#### Drug efflux pump

P-glycoprotein (P-gp), also known as multidrug resistance protein 1 (MDR1), is an ATP-dependent efflux pump that has been implicated in ALK-independent resistance^[80]^. In one study of 11 patients with ALK-positive NSCLC with acquired resistance to ALK TKI, the overexpression of P-gp was observed in three cases^[80]^. Later studies have also suggested that P-gp mediates the poor CNS penetrance of crizotinib and ceretinib, as both are P-gp substrates^[80,81]^. This is further supported by the increased CNS penetrance seen with alectinib, which is not a P-gp substrate^[82]^.

## ACQUIRED RESISTANCE TO CRIZOTINIB

Although crizotinib transformed the management of advanced, ALK-positive NSCLC, patients still experience disease progression, most commonly within 1-2 years of crizotinib treatment^[9,13]^. Early efforts to identify molecular mechanisms of resistance were focused on the identification of on-target mutations. In one small series of patients with acquired resistance to crizotinib, on-target mutations were identified in five of eighteen patients (27.8%)^[47]^. Across all studies, approximately 30% of ALK-positive NSCLC patients with acquired resistance to crizotinib exhibited ALK-dependent mechanisms of resistance. The most common mutations identified within the ALK tyrosine kinase domain are L1196M and G1269A. Additional secondary mutations associated with crizotinib resistance include C1156Y, G1202R, I1171T/N/S, S1206C/Y, E1210K, L1152P/R, V11180L, I1151T, G1128A, and F1174V [Table 2]^[83-86]^.

Although on-target ALK mutations have been well-documented in crizotinib resistance, 50% of patients lack these genetic alterations, suggesting significant ALK-independent resistance and modest benefits of next-generation ALK inhibitor use. In such cases, bypass signaling pathways (EGFR, HER2/3, protein kinase C) have been frequently implicated^[59]^. To address the challenges of crizotinib resistance, several second-generation ALK TKIs have since been developed. These are associated with increased selectivity, potency, and CNS penetration. To date, three second-generation TKIs have been approved by the United States FDA for the management of crizotinib-resistant, ALK-positive NSCLC: ceritinib, alectinib, and brigatinib. Ceritinib was approved on the basis of the ASCEND-1 trial, a phase I study that demonstrated a median PFS of 6.9 months and an ORR of 56% in patients previously treated with crizotinib^[17]^. The use of alectinib following treatment with crizotinib was approved following two single-arm studies, NP28673 and NP28761, which demonstrated a median PFS of 8.1 to 8.9 months and an ORR of 48% to 50%^[25,26]^. In a randomized phase II trial (ALTA), another second-generation ALK TKI, brigatinib, demonstrated a median PFS of 15.6 months and an ORR of 62%^[31]^.

Despite being developed in the post-crizotinib setting, second-generation ALK TKIs have since replaced crizotinib as the preferred frontline therapy. In a randomized phase III trial (ALEX), frontline alectinib demonstrated significant prolonged PFS compared to crizotinib (25.7 *vs*. 10.4 months). The ALEX trial employed an independent committee to evaluate CNS-related effects, finding a significant benefit in the alectinib arm. Interestingly, the alectinib arm seemed to demonstrate these benefits while having a comparable safety profile to crizotinib. These data contributed to the shift toward using second-generation ALK TKI as a first-line agent. One factor to be considered about this trial is the generalizability of the results. For example, participants in this study were characterized as “Asian” and “Not Asian.” An ethnic breakdown of the cohort, in addition to age analysis, would help detail this article’s external validity^[27]^.

At the time of this publication, the FDA recently accepted a new drug application for a novel second-generation ALK TKI for patients with metastatic ALK-positive NSCLC. Ensartinib (S-396) is a novel, second-generation TKI with additional activity against MET, ABL, ROS1, LTK, aAxl, and EPHA2^[87]^. In a phase 1 to phase 2 trial, ensartinib was associated with a response rate of 60% and a median progression-free survival of 9.2 months in ALK-positive efficacy evaluable patients, with high CNS penetration^[88]^. In patients who had never received prior ALK TKI, the RR was 80% and the median PFS was 26.2 months^[88]^. In the phase 3 eXalt3 study, ensartinib showed superior efficacy to crizotinib in both systemic and intracranial disease, raising the possibility of a new first-line option for patients with ALK-positive NSCLC. The median PFS with ensartinib was significantly longer than with crizotinib [25.8 (range, 0.03-44.0 months) *vs*. 12.7 months (range, 0.03-38.6 months); hazard ratio, 0.51 (95% CI, 0.35-0.72); log-rank *P* < 0.001]. Intracranial response rate associated with ensartinib was 63.6% with ensartinib and 21% with crizotinib^[87]^.

## ACQUIRED RESISTANCE TO CERITINIB, ALECTINIB, AND BRIGATINIB

While second-generation ALK inhibitors are more potent than their first-generation counterparts, they also induce more resistance^[89]^. The mutations leading to such resistance are numerous and yet to be fully categorized. Notably, different mutations are associated with unique members of the second-generation ALK inhibitors. However, G1202R should be highlighted as a common shared acquired mutation and stands as the most prominent mechanism to induce resistance in second-generation ALK inhibitors. In one paper that analyzed mutations of patients who progressed on ALK inhibitors, G1202R mutation was found in 21% of patients who progressed on ceritinib, 29% of alectinib, and 43% of brigatinib^[44]^. Notably, G1202R mutation was only found in 2% of crizotinib specimens, highlighting its increased presence within the second generation. The G1202R mutation is a solvent front mutation that induces resistance through changes in physical conformation [Figure 1]. The mutation is located within the binding region of ALK, and the mutation results in a bulkier side chain. This results in decreased binding affinity between ALK inhibitors and the ALK region, leading to decreased efficacy of medication and progression of disease^[44]^.

In one study, F1174 C/L mutation accounted for 16.7% of mutations found after progression with ceritinib^[44]^. F1174 resides near the C-terminus of ALK’s binding site. Like G1202R, mutations in F1174 C/L decrease the affinity between ALK and certain ALK inhibitors, inhibiting treatment efficacy. F1174 mutations are also associated with increases in affinity between ALK and ATP. This upregulates the rate of ALK phosphorylation, leading to enhanced gene activation, and heightening downstream effects^[44,90]^. Other mutations or cellular changes associated with resistance to ceritinib include c-MET gene amplification, G1202R mutation, L1196M mutation and p-glycoprotein overexpression, G1202R + F1174V comutation, I1171N + F1174I comutation, I1171N + L1198H comutation, and L1196M + G1202R comutation [Table 2]^[84,91]^.

In a study examining seventeen patients following progression after receiving alectinib, mutational analysis revealed a frequency of I1171T/S mutations at 12%^[44]^. The pro-oncogenic effect of ALK I1171 mutations has been corroborated elsewhere. In another study examining ALK 117T1 mutation in neuroblastoma, an association was found between the mutation and ligand-independent activation in ALK, causing an increase in neurite growth and downstream signaling^[92]^. The patient managed to achieve remission with ceritinib, which is consistent with literature suggesting ALK mutations’ susceptibility to this medication^[93,94]^. Other mutations associated with resistance to alectinib include V1180, W1295C, L1196M, and G1202 R/L [Table 2]^[44,94,95]^.

The E1210K mutation accounted for 29% of mutations found after post-brigatinib progression in the aforementioned study^[42]^. It has also been shown to have a propensity to form compound mutations under certain circumstances; taking brigatinib after crizotinib can result in mutant subclones such as E1219K + S12016C or E1210K + D1203N^[96]^. Other mutations known to be resistant to brigatinib include D1203N and S1206Y/C [Table 2]^[97]^.

## ACQUIRED RESISTANCE TO LORLATINIB

Lorlatinib is a third-generation ALK tyrosine kinase inhibitor, which was designed to overcome resistance against earlier-generation ALK TKI. First, it was designed to have activity against the ALK on-target mutation, G1202R^[98]^. Additionally, it is highly selective for the ALK/ROS1 driver mutations^[99]^. Lastly, lorlatinib was formulated to circumvent the P-gp protein, thereby increasing its concentration within the CNS and enabling superior penetrance compared to prior ALK TKI^[75]^. However, due to this heightened CNS activity, this drug results in a side effect profile of mood disturbances, cognitive impairment, and personality changes, which may be troublesome for patients to endure, particularly during the early stages of treatment^[100-102]^. Due to the ORR of 47%, lorlatinib received accelerated FDA approval for use as a 2nd/3rd line therapy^[103]^. Subsequently, there was a phase 3 trial (CROWN trial) that compared lorlatinib to crizotinib in the first-line setting. Through this study, they demonstrated that lorlatinib had a significantly longer PFS (HR - 0.28) and a reduction in CNS progression (HR - 0.07), leading to its approval as first-line therapy, commencing in 2021^[36]^; thus, there is particular interest in data concerning long-term survival and safety. Of note, the authors of the CROWN trial acknowledged that compared to when the trial started, the practice had shifted away from using crizotinib to second-generation ALK TKI as first-line agents. Thus, the applicability of this trial is not as prominent as before. Nonetheless, it remains an important, high-quality study that contributed to lorlatinib’s approval as a first-line agent.

Despite the potency of third-generation ALK TKIs, acquired resistance remains a challenge. However, the overall frequency of on-target resistance mutations is much lower (25%-30%) than in first- and second-generation ALK TKIs, attributing to their high potency^[99]^. The majority are compound mutations, such as C1156Y + L1198F, G1202R + L1196M, I1171N + D1203N, which involve two or more mutations in the kinase domain [Table 2]^[59,96,104-106]^. Whole-exome sequencing and analysis of clonality of serial biopsy samples have shown a stepwise accumulation of mutations following sequential use of next-generation ALK TKI, with ALKG1202R- or ALKI1171N-based compound mutations being the most common. These compound mutations pose significant challenges due to complexity and are often refractory to all approved ALK TKIs, highlighting the need for alternative therapies. Of note, some compound mutations that have been implicated in lorlatinib resistance have been associated with re-sensitization to first- or second-generation ALK TKI, further adding complexity to this picture^[58]^. Off-target mechanisms of resistance have also been identified following the use of lorlatinib. 22% of patients with disease relapse on lorlatinib were associated with MET amplification^[64]^.

### Intrinsic resistance

Intrinsic or primary resistance is defined as the initial lack of response to an ALK TKI. This type of resistance is seen in 5%-7% of patients after crizotinib, 9% of patients after ceritinib, and 25% of patients after lorlatinib use^[44]^. Currently, the development of primary resistance is poorly understood. It is thought that the mechanisms that lead to acquired resistance may cause primary resistance in treatment-naive patients if already present^[44]^. Prior studies have also evaluated alternative mechanisms that may explain the development of primary resistance to ALK; Bcl-2-like protein 11 (BIM-11) with missing polymorphisms and MYC amplification have been two of the proposed alterations contributing to primary resistance to ALK inhibitors^[107,108]^.

One proposed idea is that fusion variants may mediate *de novo* resistance to ALK-targeted therapy. A prior study showed that sensitivity to ALK TKIs was correlated to the EML4-ALK fusions variants, which was thought to be due to the protein stability^[109]^. Another study demonstrated better disease control with crizotinib in patients with EML4-ALK variant 1 compared to other variants^[110]^. The fusion variants of ALK may be pivotal in assessing resistance development and potential treatment options further.

Another factor that may contribute to the development of primary resistance is the complex genetic composition of some tumors. This leads to the inaccurate detection of mutations, resulting in false positive ALK cases, which may explain a lack of response to initial targeted therapy^[111]^. This is why one should utilize different diagnostic modalities to detect positive ALK mutations prior to initiation of therapy.

Lastly, it is possible that mutations lead to the development of primary resistance to ALK TKI in a similar manner to the T790M mutation in EGFR-positive cases^[112]^. However, there have been limited data in the literature thus far on the presence of de novo ALK mutations found in ALK-positive NSCLC^[113]^. Further research should be conducted to evaluate if these de novo ALK mutations play a role in primary resistance.

## ALTERNATIVE STRATEGIES TO OVERCOME ALK INHIBITOR RESISTANCE

### Immunotherapy

Although immunotherapy has been instrumental in the treatment of NSCLC patients without actionable mutations, it has been underwhelming thus far when studied in ALK-positive patients^[44]^. Prior studies have shown that ALK-positive patients have a lower overall response rate and shorter PFS compared to ALK wild-type patients after receiving immune checkpoint inhibitor monotherapy^[44,114,115]^. Other studies have attempted to evaluate the possibility of combining ALK TKI and immunotherapy. Unfortunately, these endeavors have yielded more adverse effects than benefits^[116-118]^. However, small sample sizes should be considered when evaluating these studies. For example, one study that analyzed thirteen participants indicated that their cohort had a higher median age than the typical population with ALK-positive NSCLC, with a higher smoking population^[116]^. These factors are important to consider when considering the success of an intervention and there is hope that immunotherapy may have a future role in overcoming ALK inhibitor resistance.

### 4th-generation ALK TKI

Two fourth-generation TKIs are in development, which have activity against single and some compound mutations associated with lorlatinib resistance in the preclinical setting. TPX-0131 is a compact macrocyclic molecule that can bind completely within the ATP binding boundary to overcome a variety of ALK resistance mutations^[119]^. These ALK resistance mutations include solvent front mutations, gatekeeper mutations, and compound mutations, specifically G1202R + L1198F, G1202R + L1196M, L1196M + L1198F, and G1202R + C1156F^[120]^. The FORGE-1 (NCT04849273) is evaluating TPX-0131 as a phase I/II clinical trial. NVL-655 is a novel ALK inhibitor created to overcome ALK resistance mutations, issue of CNS toxicity, and brain metastasis. Due to its success in the preclinical setting with different ALK fusion partners, EML4 variants, and tumor contexts, NVL-655 is undergoing further evaluation in the phase I/II ALKOVE-1 trial (NCT05384626)^[120]^. The development of the new generation of ALK TKI will be critical in the field of targeted therapy as it aims to overcome the ongoing challenges of current targeted therapies.

### Combination therapies

As we discover more about off-target resistance mechanisms that more frequently occur with newer-generation ALK TKIs, this may serve as a pathway for combination therapies for currently FDA-approved targeted therapy. As previously mentioned, MET amplification is one of the pathways of off-target resistance and retrospective analysis has shown some modest benefits in combination lorlatinib + crizotinib and alectinib + capmatinib in MET amplification patients^[121]^. Another pathway of consideration, as previously mentioned, is the MAPK pathway and the use of MEK inhibitors; there have been studies on the use of MEK inhibitors with ALK TKI, but the study involving brigatinib + binimetinib was closed due to low accrual (NCT04005144), and another study involving alectinib + cobimetinib has had slow accrual as well (NCT03202940). Finally, SHP2 inhibitors are being studied in early phase studies in solid tumors and thus combination with ALK inhibitors may be of benefit, as there is an ongoing study with lorlatinib and SHP2 inhibitor TNO155 (NCT03202940). These combinations may help improve the efficacy and duration of response to ALK inhibitors.

### Antibody-drug conjugates

An important newer class of drugs under development for NSCLC treatment are antibody-drug conjugates (ADCs). It consists of a monoclonal antibody attached to a cytotoxic drug via a chemical linker. Once the ADC binds to a target antigen of a cancer cell, it undergoes endocytosis and is internalized within the cell. Then, the ADC will fuse with a lysosome where the antibody and the cytotoxic drug (payload) are separated, which results in the release of the payload that leads to cancer cell death^[122]^. While there have not been direct targets for ALK, some of the target pathways do involve ALK-independent resistance patterns. For example, telisotuzumab vedotin (Teliso-V) targets c-MET and has been shown to have an ORR of 53% in nonsquamous EGFR mutant NSCLC patients with c-MET overexpression^[123]^. Another key target in NSCLC is trophoblast cell-surface antigen (Trop2), which is overexpressed in over 60% of adenocarcinomas and 75% of squamous cell carcinomas in NSCLC^[124]^. One of the Trop2 ADCs is datopotomab deruxtecan (Dato-DXd), which consists of an anti-Trop2 IgG1 monoclonal antibody covalently linked to a topoisomerase I inhibitor^[124]^. TROPION-Lung05, a phase 2 study of patients with advanced NSCLC with actionable genomic alterations that progressed after targeted therapy and platinum-based chemotherapy, showed promising results with an ORR of 35.8% (95% CI 27.8-44.4) with a median PFS of 5.4 months (95% CI 4.7-7.0). Among 34 ALK-rearranged patients, the ORR was 23.5% (95% CI 10.7-41.2), with a median PFS of 4.3 months (95% CI 2.6-6.9)^[124]^. As the complexity of the acquired resistance mutations increases and many patients do not have a known acquired resistance mutation with TKI, ADCs provide an appealing solution for a therapy that could bypass these resistance mechanisms.

### Proteolysis-targeting chimeras

Proteolysis-targeting chimeras (PROTACs) are heterobifunctional molecules that utilize the endogenous intracellular proteolysis mechanism via the ubiquitin/proteasome system to degrade target proteins^[125]^. A PROTAC consists of two ligands connected by a link that binds to the protein of interest and an E3 ubiquitin ligase, resulting in ubiquitination and protein breakdown^[126]^. PROTACs are being explored to combat resistance in patients who develop on-target resistance mechanisms. In this approach, ALK can still be targeted via protein degradation despite the presence of on-target resistance mutations. The first ALK-targeted PROTAC was reported by the Gray group which showed that this compound degraded ALK but also non-specifically degraded other kinases^[127]^. Since then, other groups have created and studied PROTACs that target the ALK mutation, which have shown promise in decreasing the concentration of ALK in preclinical settings^[128-135]^. Although more research is needed, PROTACs serve as a promising alternative strategy to treat on-target resistance to ALK TKI.

### Targeting drug-tolerant persister cells

The presence of drug-tolerant persister (DTP) cells is thought to contribute to resistant disease in patients after receiving ALK TKI. When cancer cells enter this persister state, they remain in cell cycle arrest, which allows them to evade cell cancer death^[136]^. These persister cells will survive the initial exposure to TKI, thereby adapting mechanisms that will lead to acquired genetic alterations, which may result in clinical relapse^[137]^. Therefore, further research is currently evaluating different methods to target persister cells.

Local consolidative approaches may be used to combat persister cells to treat areas of residual disease after initiation of TKI^[138]^. One study is examining the efficacy of stereotactic body radiation therapy after initial TKI therapy in metastatic NSCLC with actionable driver mutations (NCT02314364). Alternative approaches to treat drug-tolerant persister cells include manipulation of the cell cycle via CDK4/6 inhibition to continue the persister state, identification of factors that maintain a persister state, and inhibition of regulatory pathways that lead to transcription^[139]^. It is important to further characterize the biology behind drug-tolerant persister cells to gain a better understanding of their role in drug resistance.

### Vaccine therapy

Another novel immune-based approach is utilizing ALK as a vaccine target to generate an immune response against ALK. This was first developed in 2015 when preclinical work showed that vaccination of transgenic mice with EML4-ALK variant 1 induced a strong immune response and prophylactically and therapeutically impaired the growth of ALK+ NSCLC tumors^[140]^. It was also discovered that there are spontaneous anti-ALK autoantibodies^[141]^. Additionally, 17% of the sample was found to have elevated levels of these antibodies^[141]^. Therefore, it is possible that an ALK vaccine could generate an anti-tumor response in patients who have the presence of these autoantibodies and may stimulate a tumor response in patients without them. More recently, a study showed that a peptide vaccine that is specific to HLA-A*02:01 and HLA-B*07:02 could prime ALK-specific CD8+ T cells to generate an immune response to ALK+ NSCLC tumors that current ALK inhibition with TKI cannot^[142]^. Further work in understanding the immune tumor microenvironment and how to prime immune cells to generate a response will be needed and may help augment TKI response [Table 3].

**Table 3:** Overview of alternative strategies to overcome ALK inhibitor resistance

**Alternative strategies**	**Description**	**Ref. and trials**
Immunotherapy	Stimulate or suppress the immune system	[44,114-118]
4th generation ALK TKI	TPX-0131: overcome ALK resistance mutations	(NCT04849273)
	NVL-655: overcome ALK resistance mutations	(NCT05384626)
Combination therapies	Combination therapy in MET amplification	[121]
	Combination of ALK and MEK inhibitors	(NCT04005144) (NCT03202940)
	Combination of ALK and SHP2 inhibitors	(NCT03202940)
Antibody-drug conjugates	Selective delivery of cytotoxic compounds to tumor cells	[122-124]
PROTACs	Heterobifunctional molecules, which utilize the cell’s intracellular proteolysis mechanism via the ubiquitin/proteasome system to degrade target proteins	[125-135]
Targeting drug-tolerant persister cells	Using local consolidative approaches to combat persister cells in areas of residual disease	[138], (NCT02314364)
	Manipulation of the cell cycle via CDK4/6 inhibition	[139]
	Identification of factors that maintain a persister state	[139]
	Inhibition of regulatory pathways that lead to transcription	[139]
Vaccine therapy	Using ALK as a vaccine target to generate an immune response that inhibits the growth of ALK-positive NSCLC	[140-142]

ALK: Anaplastic lymphoma kinase; TKI: tyrosine kinase inhibitor; MET: mesenchymal epithelial transition factor receptor; MEK: mitogen-activated protein kinase; SHP2: SH2 containing protein tyrosine phosphatase 2; NSCLC: non-small cell lung cancer.

## DISCUSSION

The treatment of ALK-positive NSCLC has shifted the treatment paradigm within lung cancer. However, treatment remains challenging due to ubiquitous molecular resistance. Since the initial discovery of EML4-ALK fusion by Soda *et al*. in 2007, many new mechanisms of acquired resistance have been identified from the use of next-generation TKI^[7]^. However, with the growing number of ALK TKIs approved for clinical use, there remain multiple considerations that need to be addressed regarding the future management of ALK inhibitor resistance.

A key consideration is the optimal sequence of ALK TKI administration, particularly when choosing the first TKI to administer. A common approach is to first utilize a second-generation ALK TKI such as alectinib, which has shown significant benefit as first-line therapy and carries a favorable side effect profile^[143]^. When choosing between second-generation TKIs, the ALTA-3 trial demonstrated no difference in PFS when comparing alectinib and brigatinib^[35]^. However, starting with a less potent ALK TKI may lead to a higher likelihood of selecting for complex compound mutations. An alternative approach is to use the highly potent third-generation ALK TKI, lorlatinib, as first-line therapy to delay the emergence of on-target resistance and prolong PFS^[144]^.

Another vital consideration when choosing a TKI is the degree of CNS penetration. ALK-rearranged NSCLC has been associated with the development of brain metastasis in 3 years in more than 50% of patients. As ALK-rearranged NSCLC patients live longer with a median OS greater than 5 years when treated with a second-generation TKI, the significance of CNS penetration is becoming increasingly important and warrants further consideration^[145,146]^. Lorlatinib has been shown to improve blood-brain barrier penetration into the CNS by downregulating secreted phosphoprotein 1 (SPP1) and inhibiting VEGF, TGF-beta, and claudin and subsequently reducing the number of tight junctions between blood-brain barrier cells. It is currently the one ALK TKI that can overcome the G1202R acquired resistance mutation^[61]^. However, lorlatinib may lead to CNS side effects that pose challenges for patients in terms of tolerance, including cognitive alterations such as mental impairment, mood effects, and visual and audio hallucinations, which are not observed with a second-generation ALK TKI^[147]^. Managing older patients and younger patients in occupations susceptible to this cognitive toxicity requires careful consideration. To date, finding an effective CNS penetrant treatment for ALK that can overcome acquired resistance without significant CNS toxicity remains an important need.

As resistance to ALK inhibitors becomes increasingly complex, another emerging strategy is to determine the sequence of ALK TKI administration based on unique resistance profiles. To do this, repeating solid tissue or liquid biopsy is important. This is demonstrated by a compelling case of re-sensitization to earlier-generation ALK TKI. A patient receiving sequential TKI for the treatment of ALK-positive NSCLC developed crizotinib resistance due to the C1156Y mutation. After treatment with ceritinib and lorlatinib, subsequent sequencing showed an L1198F mutation, which conferred resistance to lorlatinib but sensitized the C1156Y mutation. This led to a positive clinical response to crizotinib^[46]^.

Beyond informing the selection of ALK TKI sequence, comprehensive genomic profiling provides additional insight when managing ALK-positive NSCLC. A liquid biopsy provides a depiction of circulating tumor DNA (ctDNA) present in the blood, which can help guide the selection of initial ALK TKI and subsequent lines of therapy upon progression. ctDNA testing may have both prognostic and therapeutic implications. In the ALTA-3 study, patients with detectable ALK fusion on baseline ctDNA were found to have significantly worse PFS (11.1 *vs*. 22.5 months)^[35]^. Additionally, in perioperative studies involving chemotherapy and immunotherapy, ctDNA clearance with neoadjuvant treatment has portended better EFS^[148,149]^. ctDNA is also particularly useful in predicting disease progression prior to clear radiographic evidence. Thus, using ctDNA as a means of monitoring and identifying evolving clones may provide guidance when thinking about switching ALK TKI therapy upon suspected progression.

The presence of different EML4-ALK variants may also influence the selection of ALK TKI. Since these patients were all treated with crizotinib, it showed that patients with variant 1 of EML4-ALK demonstrated a better PFS compared to those with other variants (HR: 0.35, 0.129-0.929, *P* = 0.05)^[111]^. This study suggested that patients with variant 1 of EML4-ALK may benefit from the initial selection of crizotinib, but this needs to be further verified. In a subsequent study of 29 patients, it was observed that patients with the G1202R resistance mutation were more likely to have variant 3 compared to variant 1 (32% *vs*. 0%; *P* < .001). Since these patients received lorlatinib, variant 3 was seen to have a longer median PFS compared to variant 1 (11.0 *vs*. 3.3 months; *P* = 0.011)^[150]^. Therefore, this study suggested that lorlatinib may provide better outcomes for patients who exhibit variant 3. It will be important to continue exploring how variants may influence the outcomes of patients on an ALK TKI. It is important to consider multiple factors (presence of resistance mechanisms, side effect profiles, CNS involvement, and EML4-ALK variants) when selecting the optimal sequence of ALK TKI and research into these variables may help the development of future ALK-targeted therapy.

Adding to this rapidly evolving landscape, there are several novel TKIs being evaluated in clinical trials. Entrectinib (RXDX-101), a next-generation ALK inhibitor with additional activity against NTRK and ROS1 rearrangements, has been well-tolerated in several phase 1 clinical trials in patients with ROS1-fusion positive NSCLC^[151]^. Repotrectinib (TPX-0005), another multi-kinase inhibitor that targets NTRK, ALK, JAK2/STAT, and Src/FAK rearrangements, has recently received FDA approval for ROS1 fusion-positive NSCLC and remains an area of interest in the management of ALK-positive NSCLC. Belizatinib (TSR-011), a dual ALK and TRK inhibitor, unfortunately, did not demonstrate significant clinical activity. In a phase 1, open-label trial of oral TSR-011 in patients with advanced solid tumors and lymphomas, 6 of 14 patients with ALK-positive who were naive to ALK TKI experienced partial response. The remaining 8 demonstrated stable disease^[152]^. Given the already competitive landscape of ALK-inhibitor drug development, further development of belizatinib was discontinued. At the time of this publication, alkotinib (ZG-0148), another multi-kinase inhibitor with demonstrated activity against *ALK* and *ROS1* gene rearrangements, is undergoing evaluation in a phase 2 clinical trial involving patients with ALK-positive NSCLC previously treated with crizotinib (NCT04211922).

Another consideration when using ALK TKI will be adapting to the expanding indications for use. For a long time, ALK TKI has been exclusively used in stage IV ALK-rearranged NSCLC cases. The ALINA trial (NCT04302025), which looked at stage IB-IIIA ALK+ NSCLC patients, showed significant DFS in alectinib used for 2 years after surgery compared to chemotherapy in both stage II-IIIA and ITT populations^[153]^. The phase II NAUTIKA1 and ALNEO studies are looking at alectinib in a neoadjuvant setting^[154,155]^. These studies could eventually lead to ALK TKI being used in the early stages of lung cancer in the future.

Furthermore, as novel combination therapies and alternate therapeutic strategies continue to emerge, choosing the optimal sequence of ALK TKI administration will become increasingly complex. Current combination therapies include adding targeted therapy against MET, MEK, mTOR and/or SHP2.

Although the use of immunotherapy in ALK-positive NSCLC has not been promising, other new classes of drugs such as ADCs and PROTACs provide additional strategies against ALK TKI resistance. Of note, ADCs are associated with significant side effects, such as interstitial lung disease and ocular toxicities, as seen in Dato-DXd^[156]^. Further studies will be necessary to assess any possible new safety signals that might occur should a patient transition from a TKI to an ADC and then consider reverting back to a TKI. With regards to PROTACs, there has been promising data showing tumor response in patients with prostate cancer^[157]^. There are now several PROTACs that have been designed based on second-generation TKI. A CBRN ligand-based ALK PROTAC was shown to cause degradation of NPM-ALK and EML4-ALK in lung cancer lines at certain thresholds^[128]^. Despite this, ceritinib-based ALK PROTACs did not show a more significant anti-proliferative effect compared to ceritinib in ALK-positive lung cancer cells^[127]^. More research is needed in this area before clinical application can be considered [Table 3].

Targeting DTP cells through local consolidative radiation therapy and CDK4/6 inhibition may provide additional approaches to ALK TKI resistance [Table 3]^[139]^.

It is important to note that traditional cytotoxic chemotherapy and radiotherapy may also play a role in managing ALK TKI resistance. In a retrospective study, platinum/pemetrexed-based chemotherapy demonstrated modest efficacy (ORR: 29.7%, mDOR: 6.4 months) in patients with ALK-positive NSCLC who developed resistance to a second-generation ALK TKI^[158]^. The goal of adding chemotherapy following treatment with ALK TKI is to target TKI-resistant tumor cells. Further research is required to better understand if chemotherapy following ALK TKI use is associated with differences in survival compared to chemotherapy with ALK TKI as initial therapy. Local ablative therapy such as stereotactic body radiotherapy can provide meaningful control of oligoprogressive disease in salvage settings. Continuation of ALK TKI while undergoing radiotherapy may increase tumor cell radiosensitivity. In a study completed by Gan *et al*., 14 patients with brain oligoprogression who received radiotherapy while taking crizotinib experienced a longer duration of exposure compared to crizotinib (28 *vs*. 10.1 months), which was associated with longer overall survival (OS) (72% *vs*. 12%, *P* = 0.0001)^[138]^.

## CONCLUSION

Up to this point, there has been a significant amount of research that has helped us gain a better understanding of the biology involved in the ALK mutation since its identification. This has led to the development of next-generation ALK TKIs, which have improved survival in patients with ALK-altered NSCLC. However, patients develop disease progression on these current therapies due to the development of ALK TKI resistance. As mentioned above, there are many mechanisms involved with the development of ALK TKI resistance, which include primary resistance, on-target ALK resistance, and off-target resistance. This issue of overcoming ALK TKI resistance is exacerbated by the heterogeneous nature of cancer, where various resistance mechanisms may simultaneously manifest across different sites of the tumor. It is also convoluted by the increasing number of new agents targeting either ALK or associated resistance pathways and the new indications for ALK TKI. Given these challenges for combating resistance to ALK TKI, it will be important to continue fostering collaboration to create new novel approaches that will help our ALK-altered NSCLC patients have better outcomes in the future. This integration has led to the development of new novel therapies that are currently undergoing further research but may show promise in the future as a potential treatment option for patients who develop resistance to an ALK TKI.

## DECLARATIONS

### Copyright

© The Author(s) 2024.
